# Characterization and phylogenetic analysis of the complete chloroplast genome of *Juncus effusus* L

**DOI:** 10.1080/23802359.2021.1926357

**Published:** 2021-05-10

**Authors:** Mengya Lu, Zhizheng Fang, Feihan Sheng, Xiaohui Tong, Rongchun Han

**Affiliations:** a School of Pharmacy, Anhui University of Chinese Medicine, Hefei, China; b School of Life Sciences, Anhui University of Chinese Medicine, Hefei, China

**Keywords:** Chloroplast, next generation sequencing, Juncaceae, *Juncus effusus* L

## Abstract

*Juncus effusus* L., a perennial herbaceous species of family Juncaceae, is distributed mainly in warm areas worldwide. We studied the complete chloroplast (cp) genome of *J. effusus* through next-generation sequencing technology. The whole cp genome contained 170,612 base pairs, with the GC ratio as 35.99%. The 70 genes annotated from the cp genome include 32 protein coding genes, eight rRNA genes and 30 tRNA genes. The genome’s large single-copy region (LSC) was 80,640 bp, with the small single-copy region (SSC) 64,718 bp, and inverted repeat (IR) 12,627 bp. Furthermore, a phylogenetic tree was generated to evaluate evolutionary relationship between *J. effusus* and relevant species. This study will be beneficial for the further understanding and application of *J. effusus*.

*Juncus effusus* is a common traditional Chinese medicine that is widely utilized to clear the heart fire (Zhao et al. 2018), benefit urination, cure insomnia, treat urinary acerbity pain, and sore mouth and tongue (Tsoupras et al. 2011). Moreover, it has antibacterial, sedative, and antioxidant effects (Hanawa et al. 2002). With its tenacious vitality, *J. effusus* does not have strict requirements for growing in nature. It is often found in river valleys and stone cracks below 1,000 meters above sea level, especially in sunny and well-drained places (Kaczmarek‐Derda et al. 2019). So far, studies on its biological characteristics, chemical constituents and gene function have been conducted intensively (Zhao et al. 2018; Arslan et al. 2019). However, the entire cp genome of *J. effusus* was not reported. In this study, we assembled the complete cp genome and analyzed the genetic composition, which provided new insights for further researches concerning *J. effusus*.

We collected fresh leaves in the campus of Anhui University of Chinese Medicine (N31°56′28″; E117°23′18″). The specimen was deposited in Herbarium of Anhui University of Chinese Medicine (HAUCM) with the voucher number 200913AH003. Genomic DNA of *J. effusus* was extracted and purified by the CTAB approach, followed by a quality assessment to make sure values of OD_260/230_ and OD_260/280_ were in the range of 1.8–2.0. The qualified DNA sample was used to construct the library and then sequenced on an Illumina platform (Gao et al. 2019). The initial raw data included adapter contamination and reads of low quality that required trimming. Software Cutadapt (v1.9.1) was adopted to produce clean data. Subsequently, Velvet (v1.2.10) and NOVOPlasty (v2.7.2) were used for contig assembly. Finally, the resultant contigs were gap-filled by GapFiller (v1-10). Gene finding and ncRNA analysis was executed by Prodigal (v2.6.3) and Cmscan (v1.1.2) against the Rfam database (Fan et al. 2020). For gene annotation, Diamond (v0.8.15) was applied to search the NR database and Blast (v2.2.28+) was used for retrieving information from KEGG (Ogata et al. 1999). Sequence of *J. effusus* cp genome was submitted to GenBank with the accession number MW366789.

Coverage for *J. effusus* cp genome was 100% and the sequencing depth was 860, suggesting the reliability of our genome data. The cp genome was 170,612 bp long with the GC ratio as 35.99%. It contained 70 genes in all, including 32 protein-encoding genes, eight rRNA genes and 30 tRNA genes. The large single-copy region (LSC) was 80,640 bp, the small single-copy region (SSC) 64,718 bp, and inverted repeat (IR) 12,627 bp.

To the best of our knowledge, this is the first time that a complete cp genome is reported from the Juncaceae family. In order to deduce evolutionary relationship between *J. effusus* and selected species, we retrieved information from 11 other plants to construct a phylogenetic tree by using MEGA X (Kumar et al. 2018). Maximum likelihood (ML) approach was adopted with Tamura-Nei substitution model (Kumar et al. 2018) and bootstrap parameter set as 1,000 replicates. The analysis data showed that *J. effusus* had close relationship with plants from Arales.[Fig F0001]

## Data Availability

The genome sequence data that support the findings of this study are openly available in GenBank of NCBI at https://www.ncbi.nlm.nih.gov under the accession number MW366789. The associated BioProject, SRA, and Bio-Sample numbers are PRJNA688017, SRR13309655, and SAMN17167432 respectively. Maximum likelihood phylogenetic tree based on the chloroplast genome sequences from 12 selected species. Values along branches refer to the percentage of replicate trees where the associated taxa clustered together.
